# Hydrogel Check-Valves for the Treatment of Hydrocephalic Fluid Retention with Wireless Fully-Passive Sensor for the Intracranial Pressure Measurement

**DOI:** 10.3390/gels8050276

**Published:** 2022-04-29

**Authors:** Seunghyun Lee, Shiyi Liu, Ruth E. Bristol, Mark C. Preul, Jennifer Blain Christen

**Affiliations:** 1 School of Electrical Computer and Energy Engineering, Arizona State University, Tempe, AZ 85281, USA; slee346@asu.edu (S.L.); shiyi.liu.1@asu.edu (S.L.); 2 Children’s Hospital of Orange County, Orange, CA 92868, USA; 3 Phoenix Children’s Hospital, Phoenix, AZ 85016, USA; rbristol@phoenixchildrens.com; 4 Barrow Neurological Institute, Phoenix, AZ 85013, USA; mark.preul@dignityhealth.org

**Keywords:** hydrocephalus, hydrogel, MEMS, 3D printing, brain implant

## Abstract

Hydrocephalus (HCP) is a neurological disease resulting from the disruption of the cerebrospinal fluid (CSF) drainage mechanism in the brain. Reliable draining of CSF is necessary to treat hydrocephalus. The current standard of care is an implantable shunt system. However, shunts have a high failure rate caused by mechanical malfunctions, obstructions, infection, blockage, breakage, and over or under drainage. Such shunt failures can be difficult to diagnose due to nonspecific systems and the lack of long-term implantable pressure sensors. Herein, we present the evaluation of a fully realized and passive implantable valve made of hydrogel to restore CSF draining operations within the cranium. The valves are designed to achieve a non-zero cracking pressure and no reverse flow leakage by using hydrogel swelling. The valves were evaluated in a realistic fluidic environment with ex vivo CSF and brain tissue. They display a successful operation across a range of conditions, with negligible reverse flow leakage. Additionally, a novel wireless pressure sensor was incorporated alongside the valve for in situ intracranial pressure measurement. The wireless pressure sensor successfully replicated standard measurements. Those evaluations show the reproducibility of the valve and sensor functions and support the system’s potential as a chronic implant to replace standard shunt systems.

## 1. Introduction

Hydrocephalus (HCP) is a chronic neurological disorder characterized by the inability to automatically adjust the drainage physiology of cerebrospinal fluid (CSF). Nearly 1 in 500 infants born in the United States suffer from hydrocephalus. CSF is a vital supporting liquid that flows through and around the cerebral cortex, functioning as (1) a “cushion” for protecting the brain and spinal cord from external shocks; (2) a “vehicle” for nutrients necessary for the brain and removing waste; and (3) a “regulator” to adjust intracranial pressures (ICP) by flowing between the cranium and spine [1]. With regard to ICP adjustment, CSF is partially drained through the arachnoid granulations (AG), which act as one-way biological valves from the subarachnoid space (SAS) to the superior sagittal sinus (SSS). In the case of hydrocephalic patients, this draining pathway of AG is disrupted, which causes an imbalance between the amount of produced and drained CSF, leading to excess accumulation of CSF in the ventricles, the subarachnoid space, and other cisternal regions of the brain [2,3,4].

The current standard treatment for HCP is the implantation of a drainage tube, termed a shunt, between the ventricles of the brain and the abdominal cavity or atrium of the heart for providing drainage of excessively accumulated CSF. These are implanted such that an outflow catheter directs CSF away from the ventricle through the skull and into a valve that controls CSF flow into another outflow catheter from the valve, which then directs CSF into distal drainage spaces [5,6].

Unfortunately, shunts in current usage have a notoriously high failure rate. Fifty percent of shunts fail within the first two years of implantation, even though the shunts are the most popular and primary treatment method for hydrocephalus. The majority of shunt complications are catheter-based occlusion failures. Catheters of an implanted shunt are exposed to blood and cellular debris, both containing proteins that bind to the shunt, which further cause macrophages and monocytes to produce growth factors. Such accumulations of debris can cause clogging of the tubing and valve. Accumulations on the outside of the tubing and valve, in turn, can attract astrocytes and microglia, potentiating an inflammatory response. Generally, occlusion-based shunt failures are caused by foreign body response, infection, and cellular growth [5,6,7,8,9].

Therefore, a catheter-less approach to reduce the overall implant surface area would be appealing to reduce these complications. Alternative shunt methods have been studied to improve current shunt systems, including MicroElectroMechanical System (MEMS)-based devices. MEMS valves have been extensively researched and developed, with fluidic valves proposed to control CSF as an implantable valve in various forms, such as cantilever, bridge, perforated membrane, or spherical ball-type valves [10,11,12,13,14,15,16,17,18]. However, critical challenges still exist, such as reverse flow leakage [10], valve deformation in long-term operations [14], valve stiction, imperfect sealing [10,13,14], etc., leading to low reproducibility and durability of the valves. To overcome these challenges, our earlier work has proposed the hydrogel check valve, meeting the two required features: (1) a non-zero cracking pressure (from 20 to 110 mmH_2_O) and (2) negligible reverse flow leakage [17,18,19]. However, the previous work suffered from a deficiency in the accurate measurement of fluid pressure across the valve as measured inside the setup and a lack of verification of the valve in realistic environments.

**Figure 1:**
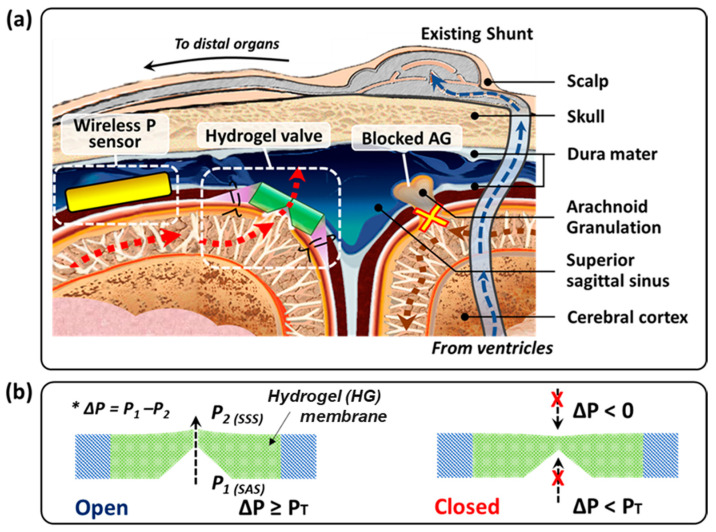
(**a**) Illustration of alternative cerebrospinal fluid (CSF) draining methods (existing shunt system and hydrogel valve of this work) for hydrocephalus treatment. Existing shunts include catheters connected from the brain ventricles to distal body spaces in order to drain CSF outwards. The proposed hydrogel valve is implanted directly in the intradural space of the superior sagittal sinus and directs CSF drainage from the subarachnoid space (SAS) into the superior sagittal sinus (SSS). This method of directing CSF allows the CSF draining process to be confined within the cranium without the use of catheters that cause many complications. (**b**) Basic operation of the valve at a cross-sectional view. When the hydrogel becomes hydrated, the swollen hydrogel structure closes the hole, forming the closed valve. When the pressure in the SAS reaches higher than the SSS over the threshold, namely, cracking pressure (P_T_), ΔP > P_T_, the swollen hydrogel valve becomes open and CSF can flow unidirectionally from SAS to SSS. When the SAS has lower pressure than the SSS by less than a differential threshold, P_T_, ΔP < P_T_, the valve is closed and blocks the CSF flow as the pressure difference cannot open the valve.

Herein, we report a physiologically and biologically realistic evaluation of the hydrogel check-valve to therapeutically manage hydrocephalus and propose a wireless measurement of fluid pressure across the valve, all designed to reduce current problems with shunt management for hydrocephalus (Figure 1). We tested the hydrogel check-valve in increasingly realistic fluidic conditions while maintaining ~37 °C during evaluation. These evaluations showed that the hydrogel check-valve maintains the functionality in each condition within the specifications of traditional shunt systems. The long-term behavior of the valve was also evaluated through automated loop functional tests, and it demonstrates improved repeatability and durability of the valve compared to our earlier work [17,18,19].

Additionally, we developed a fully-passive wireless pressure sensor to measure the pressure applied to the valve. Currently, clinical diagnosis of HCP is based on symptoms such as headache, sleepiness, vomiting, etc., which could be unrelated to HCP if there are other disease processes or comorbidities. Brain imaging usually shows the increased ventricular size, but not always. The intracranial pressure may be measured, and for those that have a valve in place, the valve may be checked for its operational status. If their HCP treatment device is malfunctioning, the ICP range will likely be abnormal, leading to HCP or collapsed CSF spaces (i.e., ventricles). In the worst case, the HCP patients will undergo surgery to interrogate their implanted device, remove it, and, if necessary, replace the valve and tubing. In order to avoid this situation, wireless pressure measurement has distinct potential as a non-invasive and non-surgical method. The fully-passive wireless pressure sensor was designed using a resistive pressure sensor and RF backscattering to transmit the ICP measurement to a receiver outside of the brain. It shows comparable output to wired pressure measurements.

## 2. Results and Discussion

### 2.1. Experimental Setup

#### 2.1.1. Bench-Top Setup

**Figure 2:**
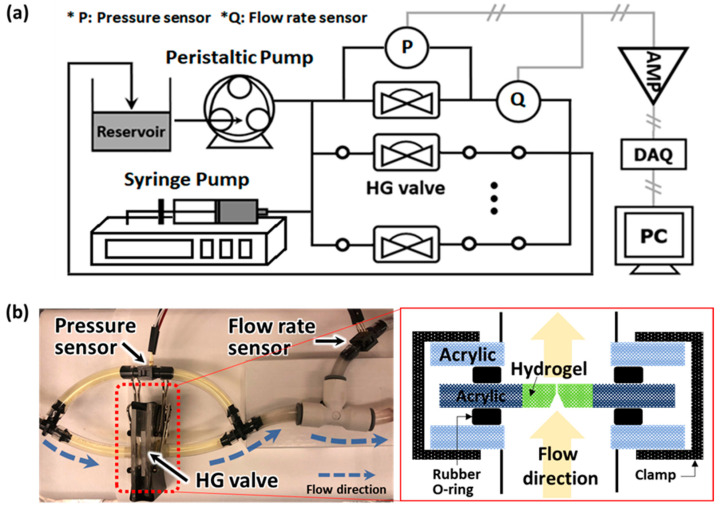
(**a**) Experimental setup for evaluation of valve functionality. For the fluid sources, a syringe pump (Model 33 syringe pump, Harvard Apparatus, Holliston, MA, USA) and peristaltic pump (P-70, Harvard Apparatus, Holliston, MA, USA) were used depending on the protocol for the experiments. Commercial resistive pressure sensors (PX26-001DV, Omega, Biel/Bienne, Switzerland) were used for measuring the pressure and flow rate with customized calibrations and the electric signals, voltage, from the sensors were transmitted through a voltage amplifier and data acquisition board (NI USB 6216, National Instruments, Austin, TX, USA) and recorded by SignalExpress software on a PC. (**b**) Photograph of the hydrogel valve setup (**left**) and diagram of the sandwich-type connecting module (**right**). The module was constructed using two acrylic plates with a hole at the center and two rubber O-rings, each placed between the acrylic plates and the respective side of the valve. The module sealing was performed by aligning the holes of the acrylic plates with rubber O-rings and valve and compressing the module on both sides of the acrylic plate using binder clips.

The hydrogel check-valve was evaluated for basic functionality in the bench-top fluidic circulatory setup. The functional tests were performed to ensure that the valve would open under forward flow and that the reverse flow leakage was properly sealed by the hydrogel swelling phenomena. The setup consists of the hydrogel valve, fluid source, and pressure & flow rate sensors (Figure 2). For the fluid circulatory system, we used a few types of source—syringe pump (Model 33 syringe pump, Harvard Apparatus, Holliston, MA, USA) and peristaltic pump (P-70, Harvard Apparatus, Holliston, MA, USA), for short-term and long-term functionality tests, respectively. Resistive pressure sensors (PX26-001DV, Omega, Biel/Bienne, Switzerland), which have 1 mmH_2_O resolution, were used for measurement of differential pressure and flow rate and calibrated for the actual experiments by a customized setting. All data were recorded by a data acquisition board (NI USB 6216, National Instruments, Austin, TX, USA) and SignalExpress 2015 software [17,18,19].

Long-term valve testing was performed to check the valve’s reliability and durability by automated loop testing setup using the programmable peristaltic pump to control fluidic flow with the number of flow cycles, flow rate, and flow direction. The valve was evaluated in a qualitative way to measure the number of valves operating instead of a quantitative way in a real-time duration due to the limits of a lab-based environment.

#### 2.1.2. In Vitro Animal Model Setup

**Figure 3:**
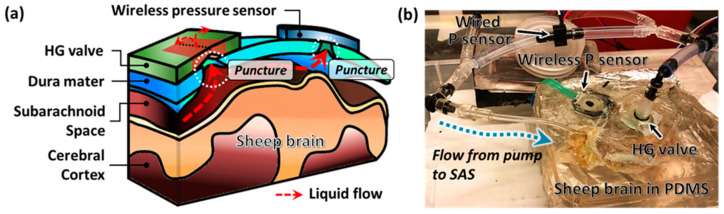
The hydrogel valve was evaluated in an in vitro setting using a fixed sheep brain. (**a**) Schematic of the experiment setup. The valve and customized wireless pressure sensor were set in the designated location on the dura mater where we manually punctured through. (**b**) Actual configuration of the experimental setup. A syringe pump injected CSF into the cavity formed by SAS and the valve. The excess portion of the manual cut was sealed by PDMS to prevent any leakage. The differential pressure was measured across the valve using conventional wired and novel wireless sensors simultaneously.

The in vitro evaluation setting for the valve was built on a fixed sheep brain (Bio corporation, Alexandria, MN, USA) (Figure 3). The sheep brain was preserved with the dura mater and enclosed by Polydimethylsiloxane (PDMS) molding to prevent fluid leakage. The hydrogel valve and customized wireless pressure sensor were placed on the locations we punched through the dura mater manually. CSF was injected into the cavity created by the valve and SAS, and the differential pressure across the valve was measured by the resistive pressure sensor.

### 2.2. Hydrodynamic Valve Characteristics

**Figure 4:**
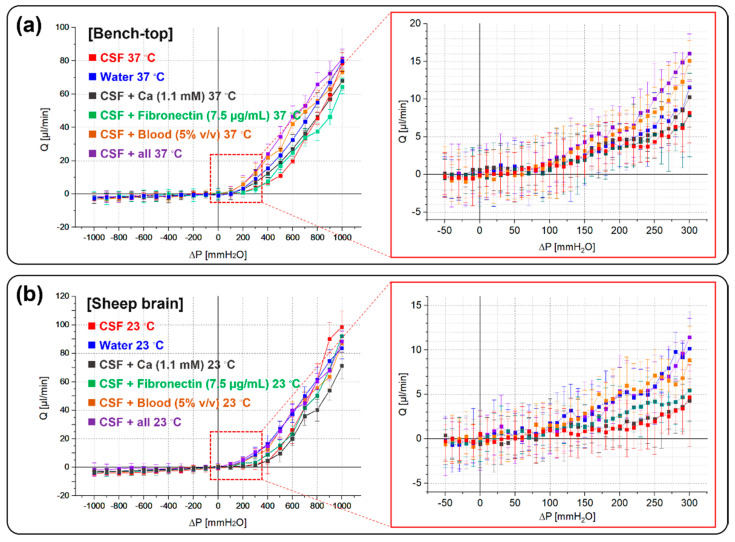
A series of increasingly worst-case fluidic conditions were used to emulate short-term conditions related to various failure modes seen in traditional shunt systems, namely occlusion due to foreign body response, fibrous encapsulation, and mineralization due to calcification. The functional tests were performed on (**a**) bench-top and (**b**) in vitro fixed sheep brain setups. The hydrogel check-valve remained functional, with a reasonable cracking pressure range and negligible reverse flow leakage. The detailed results of the valve’s functionalities are described in Table 1.

The basic hydrodynamic response of the hydrogel valve was measured and displayed the valve operation within the target range in terms of normal CSF drainage in bench-top testing (Figure 4a) and in an in vitro fixed sheep brain (Figure 4b). To evaluate the valve in more realistic patient conditions, the valve was tested in the emulated biological fluids, all based on CSF with various additives known to generate occlusion-based failures in traditional shunts: Water, CSF, CSF + Calcium (1.1 mM), CSF + Blood (5% *v*/*v*), CSF + Fibronectin (7.5 μg/mL), and CSF + All additives (Calcium + Fibronectin + Blood) at ~37 °C [20,21,22]. These additives were used to simulate worst-case scenarios such as fibrous encapsulation, excessive foreign body response, and device calcification: all situations where current shunts are known to fail. In general, the implanted shunt is stained with blood and cellular debris because the brain tissue and blood barrier are damaged during shunt implantation. The proteins from the blood and cells can bind to shunt-induced macrophages and monocytes to produce growth factors and consequently attract astrocytes and enhance the inflammatory response. Mineralization or calcification cases are also often mentioned in studies of shunt failure. This results in mechanical stress and barium sulfate added to the catheter causing nucleation of mineral deposits, ultimately leading to disintegration and the cracking of the catheter. The respective concentrations for each additive were based on concentrations from studies analyzing patient CSF [23,24,25].

Note that many of these tissue-based occlusion mechanisms are not tested, just the direct response of the hydrogel valve to the fluid and additives. The hydrogel valve remained functional, showing high diodicity without a noticeable reverse flow leakage while being tested under the six fluid conditions. The measured average P_T_ and leakage flow were 56.6 ± 5.4 mmH_2_O and 1.55 μL/min in the bench-top experiments and 115.3 ± 6.2 mmH_2_O and 2.0 μL/min in the sheep brain experiments. The measured P_T_ from the sheep brain experiments is higher than that of the bench-top evaluation. This may be because the sheep brain is deformable (higher compliance), and the valve was placed on the thin flexible dura mater, possibly leading to reduced pressure across the valve [19]. Detailed measurements of the valve’s flow response are shown in Table 1.

### 2.3. Fully-Passive Wireless Pressure Sensor

**Figure 5:**
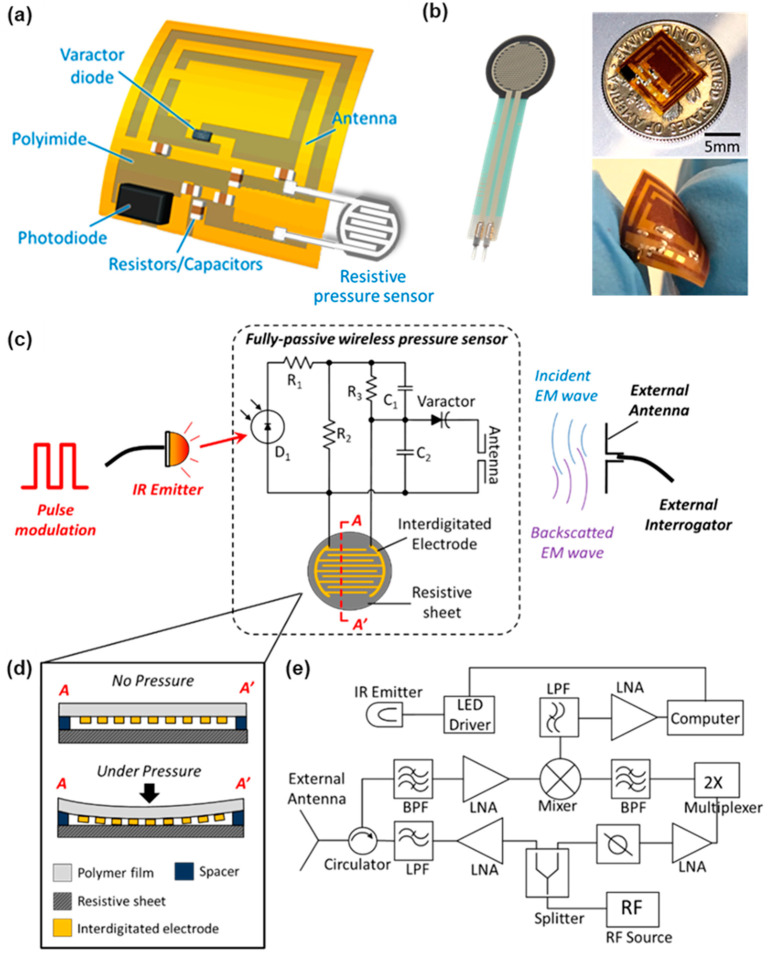
Wireless intracranial pressure (ICP) monitoring through a fully-passive method. (**a**) Illustration of the fabricated wireless fully-passive pressure sensor. (**b**) Photograph of the thin film force sensing register (FSR 402, Interlink Electronics, Camarillo, CA, USA) used for the resistive pressure sensor (Left) and the wireless fully-passive pressure sensor fabricated on a flexible polyimide substrate (Right). (**c**) Overall view of the system operating principle. Wireless fully-passive pressure sensing is accomplished using both infrared (IR) and radiofrequency (RF) electromagnetic energy. The photodiode (D_1_) on the sensor receives the modulated IR light from the external LED IR emitter and generates an electrical voltage signal, which has the same waveform as the external modulation pulse. The generated pulse is voltage divided by the resistors R_1_–R_3_ and the pressure-sensitive resistor (**d**), and outputs to the varactor diode. The voltage across the varactor diode is mixed with incident RF energy and wirelessly transmitted to the external interrogator using the RF backscattering method. The external interrogator then extracts the pressure information through a series of demodulation and calculation processes. (**d**) Operation principle of the pressure-sensitive resistor. The resistor comprises a pair of interdigitated electrodes which are printed on a flexible polymer film. The polymer film is placed on top of a resistive sheet with a spacer sandwiched between. Under external pressure, the polymer film deforms and results in contact between the interdigitated electrodes and the resistive sheet, lowering the resistance of the resistor. (**e**) Detailed structure of the external interrogator for extracting the pressure value.

Wireless monitoring of intracranial pressure (ICP) was successfully accomplished in a completely passive manner. The overall operating principle of the wireless fully-passive pressure sensor is demonstrated in Figure 5a. Herein, two different kinds of energy, namely infrared light (IR) and radiofrequency (RF) electromagnetic wave, are adopted to enable the wireless acquisition of pressure. An external LED IR emitter radiates a beam of IR light whose intensity is modulated by an external pulse signal to enable chopping. The photodiode D_1_ on the sensor senses the variation of IR energy and converts it to an electrical voltage signal, which has the same waveform as the external pulse. The generated voltage signal is divided by a voltage divider circuit consisting of resistors R_1_-R_3_, capacitor C_1_, and the pressure-sensitive resistor. Figure 5b shows the structure of the pressure-sensitive resistor. A pair of interdigitated electrodes is printed on a flexible polymer film. The film is placed upside down on top of a resistive sheet, with a spacer sandwiched in the middle. Under external pressure, the flexible polymer will deform, causing the interdigitated electrodes to contact the resistive sheet, thereby reducing the overall resistance between the two interdigitated electrodes. This forms a pressure sensing resistor whose resistance decreases as external pressure increases. As a proof-of-concept prototype, the pressure-sensitive resistor is assembled using a commercially available force-sensitive resistor (FSR). The polymer film with interdigitated electrodes is removed from the commercial FSR and is adhered to the resistive sheet (Velostat) using double-sided tape, which also functions as a spacer. Assembled pressure-sensitive resistor is sealed with sealing tape. Two electrical leads connect the pressure-sensitive resistor to the fully-passive wireless sensor.

The voltage divider circuit measures the resistance of the pressure-sensitive resistor and outputs an electrical signal to the varactor diode. Thus, the external pressure value can be obtained by measuring the amplitude of voltage signal, *V*_m_, across the varactor diode, which is accomplished utilizing RF backscattering [26]. In short, the external interrogator generates and radiates an RF electromagnetic wave (*f*_0_). The sensor receives the incident EM wave through its integrated antenna. The varactor diode then mixes the RF signal (*f*_0_) with the target signal (*f*_m_) and produces the third-order mixing products (2*f*_0_ ± *f*_m_), which are scattered back and picked up by the external interrogator. The external interrogator extracts the target signal (*f*_m_) through a series of filtering and demodulating procedures. The extracted signal is then processed by a computer to calculate the pressure value. The fully-passive wireless sensor is fabricated using a copper-clad polyimide pad, and discrete surface mount electronic components, including photodiode, varactor diode, resistors, and capacitors, are soldered onto the exposed pad.

### 2.4. Wireless Pressure Measurements

The pressure measurements were also performed by using a wireless pressure sensor. The wireless pressure sensor required unit calibration first with arbitrary units since the pressure to resistance relationship is highly fabrication dependent. The wired pressure sensor was used for the calibration as a reference measurement, while the wireless pressure sensor measured the pressure variance. The relationship between the wireless and wired sensor showed linear behavior in the positive pressure range and revealed a slope of 0.0012 a.u./mmH_2_O with a coefficient of determination value of R^2^ = 0.9687, obtained from a fitted linear regression model. In the negative pressure range, the wireless pressure sensor could not measure the pressure variance due to its geometrical limit of resistance. The sensor can measure pressure only in one direction currently; bi-directional wireless pressure measurement will serve as the subject of future studies. 

**Figure 6:**
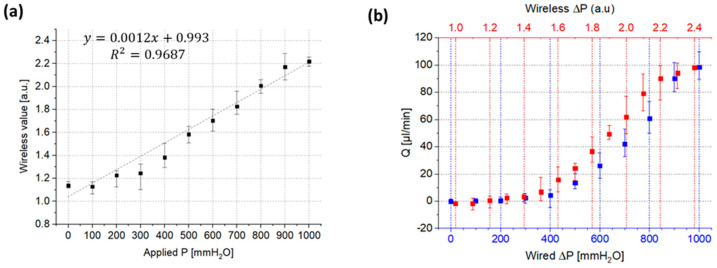
The calibrated wireless pressure sensor was used for valve functional tests in the positive pressure range and served to provide reasonable hydrodynamic behavior of the valve. (**a**) Calibration of the wireless sensor in terms of the applied pressure. (**b**) Flow response of the valve in the wired/wireless measurement. The valve demonstrated reasonable flow response in both modes with high flow diodicity and no flow leakage. By the wired measurement, the valve shows flow rates of 0~98.5 µL/min, P_T_ of 113.0 ± 9.8 mmH_2_O, and negligible reverse flow leakage of 3.7 µL/min. The wireless measurement also displays that the valve has a comparable flow response to the wired measurement with a P_T_ of 108.1 ± 3.4 mmH_2_O.

The calibrated wireless pressure sensor was used for valve functional tests in sheep brain experiments, and the results shown in Figure 6 display reasonable hydrodynamic behavior of the valve within the target specification. The valve has a P_T_ of 108.1 ± 3.4 mmH_2_O, which is clearly comparable to the result of wired measurement, a P_T_ of 113.0 ± 9.8 mmH_2_O.

### 2.5. Long-Term Functional Tests

**Figure 7:**
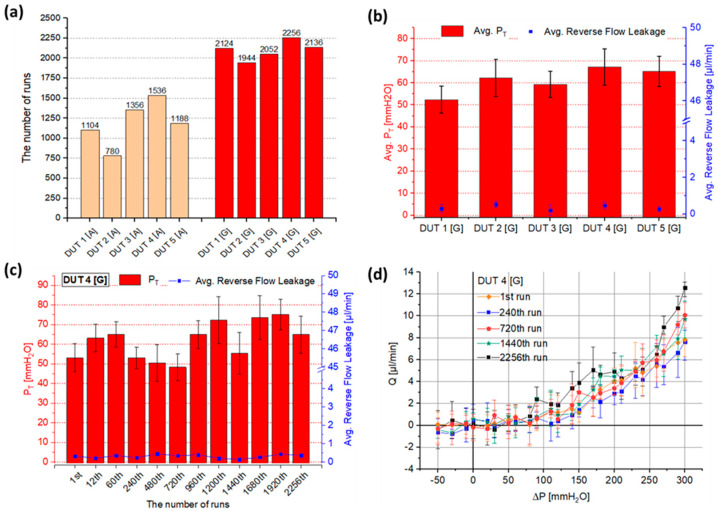
Automated repetitive tests were performed by a programmable peristaltic pump to evaluate the valve’s long-term functionality. In order to enhance the reliability and durability of the valve, glass was used for the surrounding substrate instead of the acrylic plate because glass has higher adhesion strength than acrylic due to its superior hydrophilicity. One cycle consists of flow response measurements in forward and reverse flow and is analogous to a one-day operation of the valve [20]. (**a**) Comparison of the automated repetitive test results between acrylic-based (DUT [A]) and glass-based devices (DUT [G]). The results of the acrylic-based devices (DUT [A]) are referenced from our previous study [19] for comparison purposes. The 4 out of the 5 devices with glass surrounding substrate (DUT 1[G], 3[G], 4[G], and 5[G]) marked much higher run cycles, >2000. Generally, glass-based devices display ~50% higher running times than acrylic-based devices. (**b**) The average value of P_T_ and reverse flow leakage of the devices during the repetitive tests. The devices show P_T_ and reverse flow leakage in the range of 52.3~67.1 mmH_2_O and < 0.5 μL/min, respectively. Error bar: standard deviation. (**c**) P_T_ and reverse flow leakage of DUT 4[G] over 2256 cycles. The measurements show the valve functions consistently at 48.3 < P_T_ < 75.1 mmH_2_O and reverse flow leakage of < 0.3μL/min. Error bar: standard deviation. (**d**) The flow response of the valve (DUT 4[G]) shows high diodicity behavior with negligible reverse flow leakage throughout 2256 runs. Error bar: min to max.

We performed repetitive functional tests to evaluate the valve’s long-term feasibility in terms of the run cycles. The test was controlled by a programmable peristaltic pump for repetitive sequences of forward/reverse cycles in the range of −50 < ΔP < 300 mmH_2_O [27]. In order to advance the reliability and durability of the valve, glass was used as the supporting material instead of acrylic. Because the hydrophilicity of glass is higher than acrylic, it provides more robust adhesion strength with hydrogel than acrylic-hydrogel. A total of 5 devices were used for the evaluation and maintained comparable cracking pressure and negligible reverse flow leakage for >2000 running times within the range of 52.3 < P_T_ < 67.1 mmH_2_O and < 0.5 μL/min, respectively (Figure 7a,b). Compared to the results of previous devices using acrylic, glass-based hydrogel valves display much enhanced long-term functionality, increased by ~50% as a function of run cycles. Throughout the repetitive testing, the device under test #4 (DUT 4[G]) out of the 5 devices remained within design specifications over the course of 2256 cycles of running with relatively constant cracking pressure and little reverse flow leakage of 48.3 < P_T_ < 75.1 mmH_2_O and < 0.4μL/min, respectively (Figure 7c).

We performed repetitive functional tests to evaluate the valve’s long-term feasibility in terms of the run cycles. The test was controlled by a programmable peristaltic pump for repetitive sequences of forward/reverse cycles in the range of −50 < ΔP < 300 mmH_2_O [27]. In order to advance the reliability and durability of the valve, glass was used as the supporting material instead of acrylic. Because the hydrophilicity of glass is higher than acrylic, it provides more robust adhesion strength with hydrogel than acrylic-hydrogel. A total of 5 devices were used for the evaluation and maintained comparable cracking pressure and negligible reverse flow leakage for >2000 running times within the range of 52.3 < P_T_ < 67.1 mmH_2_O and < 0.5 μL/min, respectively (Figure 7a,b). Compared to the results of previous devices using acrylic, glass-based hydrogel valves display much enhanced long-term functionality, increased by ~50% as a function of run cycles. Throughout the repetitive testing, the device under test #4 (DUT 4[G]) out of the 5 devices remained within design specifications over the course of 2256 cycles of running with relatively constant cracking pressure and little reverse flow leakage of 48.3 < P_T_ < 75.1 mmH_2_O and < 0.4μL/min, respectively (Figure 7c).

## 3. Conclusions

The hydrogel check valve presented here provides near-identical flow characteristics with an above zero cracking pressure and little to no reverse flow leakage as an alternative HCP treatment method. Ultimately, we have developed an implantable valve which is capable of accurately replicating the function of arachnoid granulations in a variety of in vitro models of hydrocephalus with biologically and physiologically realistic conditions.

The hydrogel swelling effect is a critical component in determining non-zero cracking pressure and countercurrent leakage, which allowed us to manufacture a valve that can meet the required parameters. Therefore, characterizing and optimizing the hydrogel in terms of stiffness, roughness, and swelling ratio and correlating it with valve performance would help improve the robustness and performance of our systems, which will remain as our future work. The wireless pressure measurement was also performed using a customized fully-passive pressure sensor. By comparison with the wired measurement, the results validate the valve’s potential for non-invasive and non-surgical ICP measurement.

## 4. Materials and Methods

### 4.1. Valve Design and Fabrication

**Figure 8:**
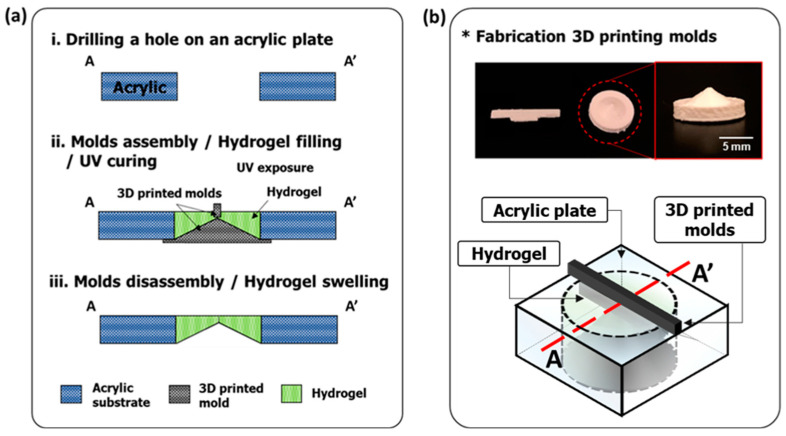
(**a**) Fabrication process: i. Drilling a hole in the center of an acrylic plate. ii. 3D printed molds are assembled in the hole, then the prepared hydrogel solution is poured and cured by UV light (365 nm, 400 mJ/cm^2^). iii. The 3D printed molds are disassembled and the device is immersed in water to swell the hydrogel. (**b**) The actual 3D printed molds and illustration of the parts assembled for the device fabrication.

The valve was designed by considering the actual SSS of the human skull for ideal future implantation; the valve needs to be comparable to or smaller than the SSS. The SSS roughly has a mean diameter of 7.3–8.8 mm [28]; thus, the valve was designed with a 7 mm diameter. The target specification of the valve was designed by referencing traditional shunt systems, which are evaluated by industry standards, ISO 7197 and ASTM F647, in terms of cracking pressure, fluidic resistance, and maximum reverse flow leakage [29,30]. Moreover, we considered the actual condition of HCP patients; the normal ICP in humans ranges from −100 to 350 mmH_2_O, and it increases to >500 mmH_2_O for hydrocephalus patients. Overall, the valve aims to operate at the range of −200 < ΔP < 600 mmH_2_O [3,8,9]. The target cracking pressure, P_T_, is 10 to 230 mmH_2_O [3,6]. The valve is composed of a hydrogel and surrounding substrate to support the valve structure. 3D-printed molds are assembled in the punctured hole at the center of the surrounding plate then the liquid-state hydrogel is cured inside the hole by UV light to be solidified (Figure 8).

#### 4.1.1. Hydrogel

Hydrogels are hydrophilic and absorbent polymeric networks which can contain over 90% water. Therefore, the mechanical properties of a hydrogel can be very similar to natural tissue; this, along with their excellent biocompatibility, has led to their frequent use in medical devices. In this study, the preparation of the hydrogel solution was performed by mixing the base (2-hydroxyethyl methacrylate, Sigma Aldrich, St. Louis, MO, USA), crosslinker (ethylene glycol dimethacrylate, Sigma Aldrich, St. Louis, MO, USA), and photoinitiator (2,2-dimethoxy-2-phenylacetophenone, Sigma Aldrich, St. Louis, MO, USA) at a ratio of 1: 0.04: 0.1, respectively [17,18,19]. This solution was poured onto the prepared substrate and cross-linked by exposure to UV of ~400 mJ/cm^2^ and a 365 nm wavelength for curing before the hydration step and immersed in water for 24 h at room temperature, 23 °C.

#### 4.1.2. 3D-Printed Mold

The tapered hydrogel structure was fabricated using 3D printed molds (Figure 8b). 3D printing implements a technology known as additive manufacturing involving iteratively depositing horizontal cross-sections of the desired 3D object [31]. This technology allows for the rapid development of custom 3D models through reducing fabrication costs and the time required to prototype custom models [32,33]. To create the models, 3D printers deposit layers of molten thermoplastic, a process called Fused Filament Fabrication (FFF) [31]. The design of these 3D models requires 3D modelling software. The models used in this research were designed in Autodesk’s Fusion 360 isometric modeling software and were printed on the Ultimaker 2 Go 3D printer (Utrecht, Netherlands) with polylactic acid (PLA) filament. The Ultimaker 2 Go has a positioning precision of 12.5 µm in the horizontal direction and a precision of 5 µm in the vertical direction.

#### 4.1.3. Cerebrospinal Fluid Preparation

CSF was collected from hydrocephalic patients at the Phoenix Children’s Hospital with an approved materials transfer agreement (MTA) and used under an Institutional Biosafety Committee (IBC) approved protocol. The CSF solutions were from an aggregate of multiple CSF samples and maintained in a 4 °C environment when not in use. Five CSF sample solutions were generated from an aggregate of patient CSF, four of which were spiked with various additives known to generate occlusions in traditional shunts: calcium (1.1 mM), fibronectin (7.5 µg/mL), blood (5%), and all three combined [20,21,22].

### 4.2. Wireless Pressure Sensor

One of the main symptoms of shunt failure is headache [4,5]. As this is a non-specific symptom with many causes, it can be difficult to determine whether this, or any symptom, is due to uncontrolled hydrocephalus. In the worst case, shunt patients will need exploratory surgery to determine whether their device is working appropriately. The most straightforward way to check the valve functionality is to observe the CSF pressure in the brain, the intracranial pressure (ICP), because ICP is the most related factor for showing HCP symptoms. If the HCP treatment device is not working properly, the ICP will be out of the normal range—usually elevated. Through ICP measurement, we can confirm that the symptoms must be due to issues with the device.

To this end, we need to observe the status of the device when the HCP patients show any related symptoms of device failure in a more patient-friendly way. Therefore, we developed a fully-passive wireless pressure sensor to measure ICP non-invasively. The sensor was designed using a resistive pressure sensor and RF backscattering to transmit the ICP measurement transcranially.

The RF components of the device use the varactor and antenna to encode information about the sensor voltage onto the second harmonic of a backscattered RF signal [26]. Additionally, the present design uses a secondary input from an external LED powering an internal photodiode. The LED and photodiode operate at infrared (IR) wavelength in order to pass through tissue. The LED is pulsed at two different frequencies in order to encode information from the pressure sensor in a manner which is resilient to natural variations in the RF and IR attenuation.

#### 4.2.1. Pressure Value Calculation

Wireless intracranial pressure (ICP) monitoring through a fully-passive method is shown in Figure 5. External pressure affects the resistance of the pressure-sensitive resistor. Increasing the pressure decreases the resistance. To read out the resistance change, *R*_3_, *C*_1_, and the resistive sensor form a voltage divider circuit, which divides the output voltage of the photodiode based on the impedance ratio between *R*_3_‖*C*_1_ and the resistance of the pressure-sensitive resistor. For simplicity, suppose the photodiode generates a sine wave to the voltage divider circuits. The sine wave has a frequency of f1 and an amplitude of *A_i_*_1_. The resistance of the pressure sensor is *R_x_*. Then the amplitude of the output signal, *A_o_*_1_, can be written as:

(1) $$A_{o1}=\frac{R_{x}}{Z_{t1}}\times A_{i1}$$

where *Z_t_*_1_ represents the impedance of *R*_3_‖*C*_1_ (the impedance of *R*_3_ in parallel with *C*_1_). For simplicity, suppose *R*_3_ is 100 kΩ and *C*_1_ is 1 nF, then *Z_t_*_1_ can be expressed as:

(2) $$Z_{t1}=\sqrt{10^{10}{\left[Im\left(\frac{1}{1+i\frac{\pi f_{1}}{5000}}\right)\right]}^{2}+{\left[R_{x}+10^{5}Re\left(\frac{1}{1+i\frac{\pi f_{1}}{5000}}\right)\right]}^{2}}$$

where *Re*(*f*) and *Im*(*f*) denote the real and imaginary parts of *f*. The amplitude of the voltage divider output signal *A_o_*_1_ is a function of the pressure-sensitive resistor (*R_x_*), photodiode output voltage *A_i_*_1_, and the modulation frequency *f*_1_. The diode output voltage *A_i_*_1_ is greatly affected by the external environment, making the output *A_o_*_1_ unstable. To overcome such an effect, a second modulation frequency, *f*_2_, is introduced. Under *f*_2_, the output signal amplitude can be written as:

(3) $$A_{o2}=\frac{R_{x}}{Z_{t2}}\times A_{i2}$$

where *Z_t_*_2_ is the impedance of *R*_3_‖*C*_1_ at *f*_2_, which can be expressed as:

(4) $$Z_{t2}=\sqrt{10^{10}{\left[Im\left(\frac{1}{1+i\frac{\pi f_{2}}{5000}}\right)\right]}^{2}+{\left[R_{x}+10^{5}Re\left(\frac{1}{1+i\frac{\pi f_{2}}{5000}}\right)\right]}^{2}}$$

The ratio between *A_o_*_1_ and *A_o_*_2_ is:

(5) $$\mathrm{Ratio}=\frac{A_{o1}}{A_{o2}}=\frac{A_{i1}Z_{t2}}{A_{i2}Z_{t1}}$$

Since the voltage output of the diode detector is not affected by the frequency, *A_i_*_1_ = *A_i_*_2_. Therefore, the ratio is:

(6) $$\mathrm{Ratio}=\frac{Z_{t2}}{Z_{t1}}$$

The above equation shows that the ratio is only a function of *R_x_* (resistance of the pressure-sensitive resistor), whose value is only related to the external pressure. Therefore, the pressure value can be obtained by calculating the ratio between *A_o_*_1_ and *A_o_*_2_.

The two frequencies (*f*_1_ and *f*_2_) are chosen to be 500 Hz and 2000 Hz, respectively. It should be noted that the actual signal outputted by the photodiode is a pulse wave instead of a sinewave; therefore, additional digital filters need to be applied during the post-processing steps. During testing, the DAQ output alternates between two frequencies (*f*_1_ and *f*_2_) of the square wave to modulate the emission of IR light. The ratio of the two signal amplitudes is measured to calculate the real-time pressure value.

#### 4.2.2. The External Interrogator

Figure 5e shows the structure of the external interrogator. The RF source (RF function generator E4432B, Agilent, Santa Clara, CA, USA) produces a 2.33 GHz RF carrier (*f*_0_) signal, which is equally divided into two paths through a power splitter [26]. The first path doubles the frequency to be 4.66 GHz (2*f*_0_) via a frequency multiplexer for the local oscillator (LO) of the down-converter. The second path amplifies and filters the RF carrier and radiates the signal through a dual-band (2.4 GHz/5 GHz) ceramic chip antenna (A10,194, Antenova, Cambridgeshire, UK). Concurrently, the antenna picks up 4.66 GHz (2*f*_0_ ± *f*_m_) backscattered third-order mixing products which carry target pressure information. The circulator isolates the backscattered signal from the RF carrier. After amplifying and filtering, the third-order mixing products (2*f*_0_ ± *f*_m_) mix with the LO (2*f*_0_) to down-convert the output to be *f*_m_. The demodulated signal (*f*_m_) goes through filtering and amplifying (SR560, Stanford Research System, Sunnyvale, CA, USA) and is sampled at 40,000 bit/s using a Data Acquisition Card (DAQ, NI-6361, National Instrument, Austin, TX, USA). The Labview (National Instrument, Austin, TX, USA) program is developed to post-process the signal and calculate the pressure value.

## Figures and Tables

**Table 1:** Summary of specifications for hydrogel valves tested in worst-case environments.

Fluid Type	Bench-Top		Sheep Brain	
	P_T_ [mmH_2_O]	Q_R_ [µL/min]	P_T_ [mmH_2_O]	Q_R_ [µL/min]
CSF	46.0 ± 7.3	1.1 ± 0.9	113.0 ± 9.8	3.7 ± 1.0
Water	51.5 ± 5.5	1.3 ± 0.7	81.2 ± 4.7	2.6 ± 1.1
CSF + Calcium	38.5 ± 5.4	1.7 ± 0.7	135.1 ± 5.1	3.3 ± 0.9
CSF + Fibronectin	68.3 ± 4.7	1.7 ± 0.7	117.5 ± 5.9	1.9 ± 0.9
CSF + Blood	62.9 ± 3.2	2.1 ± 1.1	152.7 ± 4.9	2.7 ± 1.3
CSF + All	72.4 ± 6.1	1.4 ± 0.5	92.2 ± 7.0	3.0 ± 0.6

(P_T_: Cracking pressure, Q_R_: Flow rate).

## Data Availability

Not applicable.
